# The magnitude of healthcare professionals' turnover intention and associated factors during the period of COVID-19 pandemic in North Shewa Zone government hospitals, Oromia region, Ethiopia, 2021

**DOI:** 10.3389/frhs.2022.918843

**Published:** 2022-12-08

**Authors:** Mengistu Benayew, Dejene Hailu, Berhanu Gizaw, Lidya Zerihun, Mehiret Zerihun, Tiliksew Liknaw, Muluken Ayenw, Rebik Shukure, Kumera Bekele, Abera Worku

**Affiliations:** ^1^Department of Nursing, College of Health Sciences, Salale University, Fitche, Ethiopia; ^2^Department of Public Health, College of Health Sciences, Salale University, Fitche, Ethiopia; ^3^Department of Nursing, College of Health Sciences, Debre Markos University, Debre Markos, Ethiopia

**Keywords:** COVID-19 pandemic, health care professionals, turnover intention, government hospitals, factors

## Abstract

**Background:**

Healthcare professional turnover and shortages are perceived as a global issue affecting the performance of healthcare organizations. Studies show that the coronavirus disease has physical and psychological effects on healthcare workers. This study assessed the magnitude of turnover intention and related factors during the COVID-19 pandemic.

**Methods:**

A hospital-based cross-sectional study of 402 healthcare professionals working in the North Shewa Zone was conducted during the COVID-19 pandemic from 1 February to 28 February 2021. The data were collected using a self-managed structure questionnaire, entered into EpiData version 3.1, and exported to SPSS version 25 for further analysis. We performed a logistic regression analysis to identify factors related to healthcare professionals' turnover intention. Finally, the data were displayed in frequency, percentage, and summary statistics.

**Result:**

From the total of 402 study participants, 363 of them were involved in the study with a response rate of 90.3%. The magnitude of healthcare professionals' turnover intention was 56.7%. Single marital status (AOR: 3.926; 95% CI: 1.961; 7.861), completion of obligatory service years (AOR: 0.287; 95% CI: 0.152, 0.542), dissatisfaction with the training opportunities (AOR: 2.407) 95% CI: 1.232, 4.701), having no established family (AOR: 2.184; 95% CI: 1.103, 4.326), dissatisfaction with organizational decisions process (AOR: 0.483; 95% CI: 0.250, 0.932), low continuous organizational commitment (AOR: 0.371; 95% CI 0.164; 0.842), dissatisfaction with professional development opportunities (AOR: 2.407; 95% CI: 1.232–4.701), and a non-conducive work environment (AOR: 2.079; 95% CI: 1.199, 3.607) were independent predictors of turnover intention.

**Conclusions:**

Our study showed that 56.7% of healthcare professionals have turnover intention. Being unmarried, lack of training opportunities, lack of established family, having completed the obligatory service years, non-conducive work environment, low continuous organizational commitment, dissatisfaction with the decision-making of the organization, and dissatisfaction with professional development opportunities of the organization all contributed to a higher rate of healthcare professionals' turnover intention.

**Recommendations:**

Healthcare organizations and other concerned bodies should create strategies that enhance the working environment, foster continuous organizational commitment, improve organizational decision-making, and provide professional development and training opportunities to lower the rate of turnover intention.

## Introduction

Following the report of the first case of Coronavirus Disease in China Wuhan Hubei Province, the World Health Organization (WHO) declared on 31 December 2019 that the new disease was a pandemic (1). Because of its widespread infectiousness and high infection rate, it has posed a serious threat to national public health (2). The COVID-19 pandemic is an extraordinary challenge for the world, and it has placed great stress on healthcare systems globally, increased the demand and workload for healthcare workers, and had an impact on the physical and mental health of healthcare workers due to fear of contracting the disease or passing it on to their loved ones (3).

Healthcare workers, the main force in the fight against the pandemic, are at greater risk than others. They developed a sense of frustration and helplessness while caring for patients with confirmed or potentially suspected for COVID-19 (4). Studies have found that during the COVID-19 pandemic, healthcare workers were experiencing physical and mental stress (5, 6), from 2.2% to 14.5% of health care workers suffered from severe stress, anxiety and depression (7). Studies also found that working in high pressure and stress full work environment makes healthcare workers more prone to fatigue, produce psychological disorders and leading to significantly higher turnover rate (6, 8).

In countries with limited resources and increased demand for personal protective equipment during the COVID-19 pandemic, the lives of healthcare workers were severely threatened (9). A large number of healthcare workers have died of COVID-19 due to a lack of personal protective equipment, while others continue to struggle with the pandemic (10, 11). Longer working times, increased patient loads, shortages of human resources, and shortages of personal protective equipment were explained by the increased turnover intention of healthcare workers during the time of the pandemic (12). Turnover intention which is defined as the probability that employees of the organization will voluntarily leave their jobs at some point in the near future was considered to be the strongest predictor of actual turnover among healthcare personnel (13). COVID-19 and the high rate of the turnover intention of healthcare workers have become major challenges and have placed a heavy burden on the healthcare system (14). Prior to the COVID-19 pandemic, research in Ethiopia reported 52.5–67.8% magnitude of turnover intention among healthcare professionals (15–18), however, studies done during the pandemic reported turnover intentions of 56.3–70.7% (19–21). This slight discrepancy might be due to the effect of the pandemic.

Sociodemographic characteristics, organizational commitment, professional development, organizational leadership style, work satisfaction, work environment, and work performance all influence healthcare workers' turnover intention (17, 22–24). It is of great significance to understand the relation between COVID-19 and turnover intention. Therefore, the purpose of this study was to assess the current situation and influencing factors of healthcare workers' turnover intention during the COVID-19 pandemic in North Shewa Zone Government hospitals.

## Methods and materials

### Study design, setting, and period

From 1 February to 28 February 2021, a hospital-based cross-sectional study was conducted at six government hospitals, namely Fiche General Hospital, Kuyu General Hospital, Muke Turi Primary Hospital, Gundo Meskele Primary Hospital, Sheno Primary Hospital, and Chancho Primary Hospital in North Shewa Zone Oromia region, among 402 healthcare professionals. North Shewa is one of the zones of the Oromia region in Ethiopia found 111 km from Addis Ababa, the capital of Ethiopia. A total of 5,045 healthcare professionals were working in these six hospitals during the study period.

#### Source population

All healthcare professionals of the six government hospitals at North Shewa Zone in 2021.

#### Study population

All randomly selected healthcare professionals including nurses, midwives, pharmacists, laboratorist, medical doctors, radiographers, surgeons, and gynecologists of the six government hospitals at North Shewa Zone were available during the study period.

#### Inclusion and exclusion criteria

The study included all healthcare professionals that were available during the data collection period and excluded those providing care for critically ill patients, non-permanent workers, and involuntary participants.

#### Sample size determination and sampling technique

A single population proportion formula was used to determine the sample size with the prevalence of healthcare professionals' turnover intention = 61.3% from a previous study conducted in the Amhara Region, Ethiopia (17) at a 95% level of confidence and 5% margin of error.


$$\begin{array}{l} n=\frac{{\left(Z\alpha /2\right)}^{2}p\left(1-p\right)}{d^{2}}\\ \frac{{\left(1.96\right)}^{2}\;0.613\;\left(1-0.613\right)}{0.05}n\;=\;365 \end{array}$$

With a 10% non-response rate, the final sample size was 402. There were 1,230 healthcare professionals in Fiche General Hospital, 1,097 in Kuyu General Hospital, 743 in Gundo Meskele Primary Hospital, 689 in Muke Turi Primary Hospital, 682 in Sheno Primary Hospital, and 604 in Chancho Primary Hospital. Proportional allocation was done to each study hospital based on the total number of healthcare professionals. Their registration book in human resources was used as a sampling frame. By using this frame, we employed a simple random sampling technique. Accordingly, 98 from Fiche General Hospital, 88 from Kuyu General Hospital, 59 from Gundo Meskele Primary Hospital, 55 from Muke Turi Primary Hospital, 54 from Sheno Primary Hospital, and 48 from Chancho Primary Hospital were included in the study.

#### Data collection tool and procedures

The data were collected by two diploma-holder nurses using a self-administered structured questionnaire developed from the review of related literature in Ethiopia (17, 18). The questionnaire had four parts. **Part one**: Sociodemographic characteristics of the study participants (age, sex, marital status, ethnicity, religion, profession, educational status, work experience, hospital type, obligatory service year, extra income sources, and salary satisfaction). **Part two:** A turnover intention measurement scale with three questions on a 5-point Likert scale ranging from strongly disagree to strongly agree where 5 = strongly disagree, 4 = disagree, 3 = no opinion, 2 = agree, and 1 = strongly agree developed from a previous study (17) was used. We recorded strongly disagree, disagree, and no opinion as “0” and agree and strongly agree as “1” and the mean was used to classify the turnover intention status of the study participants.

**Part three** is about organizational related factors. Under this variables such as the level of satisfaction with leadership style, professional opportunity, communication level, involvement in decision making, perceived organizational support, and training opportunity in the organization were measured with yes/no questions. The level of organizational commitment was measured with questions on a 5-point Likert scale ranging from strongly disagree to strongly agree where 5 = strongly disagree, 4 = disagree, 3 = no opinion, 2 = agree, and 1 = strongly agree. Organizational commitment has three domains: continuous, affective, and normative commitment. The normative commitment was measured by three questions whereas affective and continuous commitment were measured by four questions (17). We recorded strongly disagree, disagree, and no opinion as “0” and agree and strongly agree as “1” and the mean was used to classify the level of organizational commitment for each domain.

**Part four** is about job related factors with five subdomains on a 5-point Likert scale ranging from strongly disagree to strongly agree where 5 = strongly disagree, 4 = disagree, 3 = no opinion, 2 = agree, and 1 = strongly agree. Job satisfaction related questions with, five item questions, Job performance related questions with three item questions, Work environment related questions with six item questions, Job stress related questions with eight item questions, and Work overload related questions with six item questions on five point Likert scale (17). We recorded strongly disagree, disagree, and no opinion in to “0” and agree and strongly agree in to “1” and the mean was used to classify study participants based on the job related factors.

#### Data quality control

To control the data quality, data collectors were trained and supervised by the principal investigators. Before actual data collection, a pretest was conducted on 5% of the sample size in the Fiche Town Health Center. The completeness of the questionnaire was checked before data entry.

#### Data processing and analysis

Data were entered into EpiData version 3.1 and exported to SPSS version 25 for analysis after being checked for correctness. A bivariable analysis was used to find variables that were significantly related. Variables with a *p*-value < 0.25 in bivariate analysis were incorporated into a multivariable logistic regression model to investigate independent factors by controlling for possible confounders. In the multivariable logistic regression analysis, an AOR of 95% CI with a *p*-value < 0.05 was determined to identify the associated factors. To characterize the study variables and factors under study, data were presented in frequency, proportions, and summary statistics.

#### Statement of ethical approval

Salale University's Ethical Review Committee provided ethical permission prior to data collection with the reference number SLU-IRB-003/2021. The North Shewa Zone Health Bureau and the study hospitals provided permission letters. Furthermore, after being informed about the study's goal, each study participant completed a written informed consent form.

#### Operational definition

##### Turnover intention

The extent to which health personnel intends to depart their organization in the near future. In the current study, it was measured with three questions on a 5-point Likert scale with 5 denoting strongly disagree, 4 denoting disagree, 3 denoting no opinion, 2 denoting agree, and 1 denoting strongly agree. Strongly disagree, disagree, and no opinion were recorded as 0 representing “not intending to leave” whereas, agree and strongly agree were recorded as 1 representing “intending to leave.” Depending on the mean score value, those study participants who scored the mean and above were regarded as intending to leave and those who scored below the mean were regarded as not intending to leave.

##### Organizational commitment

An organization member's psychological attachment toward their working organization. It has three domains: **Affective commitment** is an employee's emotional attachment toward their organization and is measured by four questions on a 5-point Likert scale with 5 denoting strongly disagree, 4 denoting disagree, 3 denoting no opinion, 2 denoting agree, and 1 denoting strongly agree. Strongly disagree, disagree, and no opinion were recorded as “0” whereas, agree and strongly agree were recorded as “1.” Depending on the mean score value, study participants who scored mean and above were regarded as having a high affective commitment denoted by “1” and those who scored below the mean were regarded as having a low affective commitment denoted by “0” respectively.

##### Continuance commitment

This is the level of commitment by employees who think leaving their organization would be costly and is measured by four questions on a 5-point Likert scale with 5 denoting strongly disagree, 4 denoting disagree, 3 denoting no opinion, 2 denoting agree, and 1 denoting strongly agree. We recorded strongly disagree, disagree, and no opinion as “0” and agree and strongly agree as “1.” Depending on the mean score value, study participants who scored mean and above were regarded as having high continuous commitment denoted by “1” and those who scored below the mean were regarded as having low continuous commitment denoted by “0.”

##### Normative commitment

This is the level of commitment of employees who feel obligated to stay in the organization and that staying is the right thing to do. It is measured by three questions on a 5-point Likert scale with 5 denoting strongly disagree, 4 denoting disagree, 3 denoting no opinion, 2 denoting agree, and 1 denoting strongly agree. We recorded strongly disagree, disagree, and no opinion as “0” and agree and strongly agree as “1.” Depending on the mean score value, study participants who scored mean and above were regarded as having high normative commitment denoted by “1” and those who scored below the mean were regarded as having low normative commitment denoted by “0.”

##### Job satisfaction

The state of health workers being satisfied by their job was measured by five questions rated on a 5-point Likert scale with 5 denoting strongly disagree, 4 denoting disagree, 3 denoting no opinion, 2 denoting agree, and 1 denoting strongly agree. We recorded strongly disagree, disagree, and no opinion as “0” and agree and strongly agree as “1.” Depending on the mean score, study participants who scored mean and above were regarded as satisfied with their job denoted by “1” and those who scored below the mean were regarded as unsatisfied with their job denoted by “0.”

##### Work environment

This is a pleasant working atmosphere and was measured by five questions rating on a 5-point Likert scale with 5 denoting strongly disagree, 4 denoting disagree, 3 denoting no opinion, 2 denoting agree, and 1 denoting strongly agree. We recorded strongly disagree, disagree, and no opinion as “0” and agree and strongly agree as “1.” Depending on the mean score, study participants who scored mean and above were regarded as having a non-conducive work environment which was denoted by “1” and those who scored below the mean were regarded as having a conducive work environment which was denoted by “0.”

##### Workload

This is the work pressure present in health institutions. It is measured with six questions rating on a 5-point Likert scale with 5 denoting strongly disagree, 4 denoting disagree, 3 denoting no opinion, 2 denoting agree, and 1 denoting strongly agree. We recorded strongly disagree, disagree, and no opinion as “0” and agree and strongly agree as “1.” By using the mean score, study participants were classified as having workload when they scored mean and above which was denoted by “1” and as not having workload when they scored below the mean denoted by “0.”

##### Job stress

This refers to work-related duties and responsibilities that become burdensome and impose unhealthy effects on the mental and physical wellness of employees. It is measured by eight questions rated on a 5-point Likert scale with 5 denoting strongly disagree, 4 denoting disagree, 3 denoting no opinion, 2 denoting agree, and 1 denoting strongly agree. We recorded strongly disagree, disagree, and no opinion as “0” and agree and strongly agree as “1.” By using the mean score, study participants were classified as having job stress when they scored at mean and above which was denoted by “1” and as not having work stress when they scored below the mean as denoted by “0.”

##### Job performance

This is the work-related activities expected of an employee and how well those activities were performed. It is measured by three questions rated on a 5-point Likert scale with 5 denoting strongly disagree, 4 denoting disagree, 3 denoting no opinion, 2 denoting agree, and 1 denoting strongly agree. We recorded strongly disagree, disagree, and no opinion as “0” and agree and strongly agree as “1.” By using the mean score, study participants were classified as having good job performance when they scored mean and above which was denoted by “1” and as having poor job performance when they scored below the mean denoted by “0.”

## Result

### Socio-demographic characteristics

A total of 363 health care professionals took part in this study, with a response rate of 90.3 percent. The research respondents' median age was 38, with a standard deviation of 4.973, ranging from 19 to 52 years. Male respondents were accounted 61.2% of the total study participants and 61.4% of the respondents were married. In addition, 36.7% of study participants were nurses and 52.1% of the respondents had a bachelor's degree (Table 1).

**Table 1:** Shows sociodemographic characteristics of study respondents at North Shewa Zone government hospitals (*n* = 363).

**Variables**	**Category**	**Response no (%)**
Sex	Male	222 (61.2)
	Female	141 (38.8)
Age	2–29	148 (40.8)
	30–38	180 (49.6)
	≥39	35 (9.6)
Marital status	Married	223 (61.4)
	Single	140 (28.6)
Ethnicity	Oromo	300 (82.6)
	Amhara	29 (8)
	Others	34 (9.3)
Religion	Orthodox	252 (69.4)
	Muslim	60 (16.5)
	Protestant	46 (12.7)
	Others*	5 (1.4)
Professional category	Nurse	134 (36.9)
	Midwife	45 (12.4)
	Pharmacist	93 (25.6)
	Laboratorist	30 (8.3)
	Medical doctors	31 (8.5)
	Others**	30 (8.3)
Professional qualification	Diploma	132 (36.4)
	BSc degree	189 (52.1)
	Masters & above	42 (11.6)
Year of work experience	< 2 years	123 (33.9)
	2–4 years	145 (39.9)
	≥5 years	95 (26.2)
Live with family	No	233 (64.2)
	Yes	130 (38.8)
Upgrading/specialized	No	179 (49.3)
	Yes	184 (50.7)
Hospital type	Primary	108 (29.8)
	General	255 (70.2)
Having established family	No	120 (33.1)
	Yes	243 (66.9)
Completing obligatory service year	No	126 (34.7)
	Yes	237 (65.3)
Having extra income sources	No	292 (80.4)
	Yes	71 (19.6)
Satisfied with current salary	No	267 (73.6)
	Yes	96 (26.4)

*Others, Jehovah, Wakefta.

**Others, Radiographers, Emergency, and general surgeons.

### The magnitude of turnover intention

The overall turnover intention among the study participants was 56.7% (95% CL: 52–62%). The mean turnover intention of healthcare professionals was 9.3 (± 3.16 SD), which is computed from the three intention measuring questions. Midwives, younger, and unmarried health professionals all indicated higher rates of turnover intention, with 77.8, 68.2, and 67.9%, respectively. Medical doctors, on the other hand, reported a lower, 32.3% rate of leave intention (Table 2).

**Table 2:** Magnitude of healthcare professionals' leave intention by age, sex, marital status, professional categories, and years of work experience at North Shewa Zone government hospitals (*n* = 363).

**Variables**	**Categories**	**Turnover intention status**		**Total**
		**Intent to leave**	**Intent not to leave**	
Age of respondents	20–29	101 (68.2%)	47 (31.8%)	148
	30–38	87 (48.3%)	93 (51.7%)	180
	≥ 39	18 (51.4%)	17 (48.6%)	35
Sex of respondents	Male	127 (57.3%)	95 (42.7%)	222
	Female	79 (56%)	62 (44%)	141
Marital status	Married	113 (50.7%)	110 (49.3%)	223
	Single	93 (66.4%)	47 (33.6%)	140
Professional categories	Nurses	78 (58.2%)	56 (41.8%)	134
	Pharmacy	53 (57%)	40 (43%)	93
	Midwifery	35 (77.8%)	10 (22.2%)	45
	Doctors	10 (32.3%)	21 (67.7%)	31
	Laboratory	15 (50%)	15 (50%)	30
	Others	15 (50%)	15 (50%)	30
Years of work experience	< 2 years	72 (58.5%)	51 (41.5%)	123
	2–4 years	69 (47.6%)	76 (52.4%)	145
	≥ 5 years	65 (68.4%)	30 (31.6%)	95

### Organizational related factors

According to the findings, the majority of respondents were dissatisfied with their organizational leadership (79.9%), professional growth opportunities (79.3%), organization's communication level (66.4%), and training opportunities (62.8%) (Table 3).

**Table 3:** Shows organizational and other related factors of the study conducted at North Shewa Zone hospitals (*n* = 363).

**Variables**	**Categories**	**Responses no (%)**
Have other job opportunity	No	87 (24)
	Yes	276 (76)
Satisfied with organizational leadership	No	290 (79.9)
	Yes	72 (19.8)
Satisfied with professional opportunity	No	288 (79.3)
	Yes	75 (20.7)
Satisfied with communication level of your organization	No	241 (66.4)
	Yes	122 (33.6)
Satisfied with involvement in organizational decision making	No	222 (61.20
	Yes	141 (38.8)
Satisfied with training opportunity	No	228 (62.8)
	Yes	134 (36.9)
Satisfied with perceived organizational support	No	203 (55.9)
	Yes	160 (44.1)

### Work related factors

The effect of work-related factors such as job satisfaction, job performance, job stress, work environment, and work overload were studied. The mean score for job satisfaction was 14.67 (±4.15), for job performance 9.63 (±2.91), for work environment 18.33 (±5.67SD), for job stress 24.2 (±6.69), and for work overload 18.71 (±6.05), respectively, which are computed from the five, three, six, eight, and six questions, respectively. According to the findings, 50.4% of healthcare professionals were dissatisfied with their jobs, 55.9% reported a hostile work environment, and more than half of respondents (54.5%) had experienced job stress (Table 4).

**Table 4:** Work–related factors of the study conducted at North Shewa Zone hospitals (*n* = 363).

**Variables**	**Categories**	**Responses *N* (%)**
Job satisfaction status	Unsatisfied	183 (50.4%)
	Satisfied	180 (49.6%)
Job performance status	Poor performance	155 (42.7%)
	Good performance	208 (57.3%)
Job stress status	Had job stress	198 (54.55%)
	Had no job stress	165 (45.5%)
Work environment status	Non–conducive	203 (55.9%)
	Conducive	160 (44.1%)
Work overload status	Had work overload	153 (42.1%)
	Had no work overload	210 (57.9%)

### Organizational commitment related factors

The overall organizational commitment of the study respondents was found to be 51.2%.

The mean score for affective, continuous, and normative commitment was 12.1 (±3.96 SD), 12.35 (±3.71 SD), and 11.84 (±3.89 SD) respectively. The study found that 52.6% and 54% of the study participants exhibited high normative and affective commitment. However, 59.8% of them had a low level of continuous commitment (Table 5).

**Table 5:** Shows organizational commitment related factors of the study conducted at North Shewa Zone hospitals (*n* = 363).

**Variables**	**Categories**	**Responses *N* (%)**
Affective Organizational	Low	167 (46%)
commitment	High	196 (54%)
Continuous Organizational	Low	217 (59.8%)
commitment	High	146 (40.2%)
Normative Organizational	Low	172 (47.4%)
commitment	High	191 (52.6%)
Overall Organizational	Low	177 (48.8%)
commitment	High	186 (51.2%)

### Bivariable and multivariable logistic regression analysis

In the bivariable logistic regression analysis, seventeen variables out of thirty-one were candidate variables. Marital status, profession, type of health facility, mandatory service year, salary, established family, professional opportunity, organizational communication level, involvement in organizational decision making, perceived organizational support, affective, continuous, and normative commitment, job performance, work environment, job stress, and work overload were the variables. In a Multivariable Logistic analysis, marital status, obligatory service year, professional opportunity, an established family, dissatisfaction with the organization's decision-making process, continuous organizational commitment, and condition of work environment showed significant association (Table 6).

**Table 6:** Shows result of multivariable logistic regression analysis of study conducted at North Shewa Zone hospitals (*n* = 363).

**Variables**	**Category**	**Turnover intention**		**COR (95% C)**	**AOR (95% CI)**	***p*–value**
		**Intent to leave**	**Intent not to leave**			
Marital status	Married	114	109	1.00	1.00	
	Single	92	48	1.833 (1.184–2.836)	3.926 (1.961–7.861)	0.001*
Completing obligatory service year	No	94	32	1.00	1.00	
	Yes	112	125	0.305 (0.190–0.491)	0.287 (0.152–0.542)	0.001*
Satisfied with professional development opportunity	No	179	109	2.919 (1.721–4.951)	2.407 (1.232–4.701)	0.01*
	Yes	27	48	1.00	1.00	
Hospital type	Primary	51	57	0.577 (0.367–0.909)	0.549 (0.269–1.121)	0.10
	General	155	100	1.00	1.00	
Having established family	No	77	43	1.582 (1.009–2.482)	2.184 (1.103–4.326)	0.025*
	Yes	129	114	1.00	1.00	
Satisfied with organizational communication	No	149	92	1.847 (1.189–2.869)	1.707 (0.913–3.189)	0.094
	Yes	57	65	1.00	1.00	
Satisfied with organization's decision–making process	No	106	116	0.375 (0,239–0.578)	0.483 (0.250–0.932)	0.030*
	Yes	100	41	1.00	1.00	
Continuous commitment	Low	104	113	0.397 (0.255–0.618)	0.371 (0.164–0.842)	0.018*
	High	102	44	1.00	1.00	
Affective commitment	Low	81	86	0.535 (0.351–0.815)	0.457 (0.206–1.015)	0.054
	High	125	71	1.00	1.00	
Work environment	Non–conducive	122	81	1.363 (0.897–2.071)	2.079 (1.199–3.607)	0.009*
	Conducive	84	76	1.00	1.00	

NB, ^*^Showed significantly associated variables in the multiple logistic regression analysis.

As a result, single healthcare professionals were nearly four times more likely than married healthcare workers to leave their current working organization (AOR: 3.926; 95% CI: 1.961, 7.861). Those who finished their obligatory service year had a 71% higher chance of intending to depart (AOR: 0.287; 95% CI: 0.152, 0.542) than those who did not complete their obligatory service year. Similarly, healthcare professionals who were dissatisfied with the professional opportunities were 2.4 times more likely than their counterparts to leave their current working organization (AOR: 2.407; 95% CI: 1.232, 4.701).

Healthcare professionals with no established family were 2.2 times more likely than those with established families to plan to leave (AOR: 2.184; 95% CI: 1.103, 4.326). Those healthcare professionals who were satisfied with their organization's decision-making process had a 51% lower likelihood of leaving their current working organization (AOR: 0.483; 95% CI: 0.250, 0.932) than their counterparts. When comparing healthcare professionals with a low continuous organizational commitment to those with high continuous organizational commitment, the odds of turnover intention were nearly 63% greater (AOR: 0.371; 95% CI 0.164, 0.842). Additionally, healthcare professionals who reported a non-conducive work environment were two times more likely (AOR: 2. 079; 95% CI: 1.199, 3.607) to want to leave their current working organization than their counterparts (Table 6).

## Discussion

Healthcare professionals are the key assets of the health system. Increasing and holding proficient and motivated healthcare workers is crucial for improving the quality of healthcare services. This study was conducted to examine the magnitude of health workers' turnover intention and associated factors. This is a significant issue because the turnover intention is the strongest predictor of actual turnover. In this study, we found that 56.7% (95% CL: 52–62%) of healthcare professionals have turnover intention. The magnitude of leave intention varied across the profession with a higher proportion (77.8%) among midwife professionals and a lower proportion among medical professionals (32.3%).

In the context of the COVID-19 pandemic, the magnitude of turnover intention in our study is significantly higher than the study finding in South Korea (8%) (25), in China (10.1%) (12), in Saudi Arabia (32.2%) (26), and in Ghana (49.3%) (27), respectively. A possible reason for this discrepancy might be from a lower perceived risk of COVID-19 due to the availability of personal protective equipment and materials, increased awareness of healthcare professionals, and measures taken against COVID-19 in these countries which are all reducing healthcare professionals' turnover intention. Compared with studies conducted in Ethiopia during the ordinary period, it is similar to the study findings in the Kafa Zone in southwest Ethiopia (56.3%) (19), and in the North Shewa Zone of the Amhara region (61.3%) (17). However, it is lower than the result of studies conducted in the North Gondar Zone at the Amhara Regional State hospitals (67.8%) (18), and in Addis Ababa in the primary public health facilities (70.7%) (21). Since the study was conducted late in the pandemic, turnover intention in this period may be lower than a long-term turnover intention in a normal period. This variation might be due to the increased awareness of the disease by healthcare professionals and due to the beginning of COVID-19 vaccinations which are reducing the turnover rate.

The study also examined various factors related to turnover intention. Accordingly, being unmarried, finishing an obligatory service year, having a non-conducive work environment, being dissatisfied with decision-making of the organization, being dissatisfied with professional development opportunities, not having an established family, and having a low continuous organizational commitment were significantly associated with healthcare professionals' turnover intention. Unmarried healthcare professionals were more likely to consider leaving intention than married healthcare professionals, which is consistent with a study result in Korea (28). This might be due to the fact that unmarried healthcare professionals may not be stable and will prefer to move out than married healthcare professionals. But this finding is contradicted by a study finding in Kafa Zone Southwest Ethiopia, where married healthcare professionals were more likely to have leave intention than unmarried ones (19).

The study showed that the obligatory service year was the other determinant factor in that healthcare professionals who finished their obligatory service year have higher odds of leave intention than those who had not completed their obligatory service year. This was supported by a study finding in the Wollega Zone of northwest Ethiopia (29). This might be explained by not completing the obligatory service year, which enforces healthcare professionals to be stable in one place. Another sociodemographic factor that predicts turnover intention was not having an established family in that healthcare professionals without established families were more likely than those with established families to consider leave intention. This is consistent with a study conducted in Amhara Region's North Gondar Zone (18). Another aspect explaining turnover intention was professional development opportunities. The higher turnover intention was reported by healthcare professionals who were dissatisfied with the professional development opportunities than by their counterparts.

A non-conducive work environment also predicts healthcare professionals' turnover intention. Healthcare professionals who reported a non-conducive work environment were more likely to have leave intentions than those healthcare professionals who reported a conducive work environment. This finding is consistent with studies conducted in Ethiopia (19, 23). As for the individual dimensions of organizational commitment that were linked with the intention to leave, healthcare professionals with low continuous commitment experienced leave intention more likely than those healthcare professionals having high continuous commitment. This finding was supported by a study finding in Addis Ababa, Ethiopia (21). The organization's decision-making process was found to affect the healthcare professionals' turnover intention. Satisfied healthcare professionals by their organization's decision-making process have 51% lower leave intention than their counterparts.

### Study limitation

Some limitations were noted in our study. First, our study data may be biased due to the entire self-report data. Second, it is difficult to explain the causal relationship between risk factors and turnover intention because of the cross-sectional study design. Third, the use of one sampling frame for all stratums leads to a lack of generalizability to the study population.

## Conclusions

In this study, more than half of the study participants reported turnover intention. Variables such as single marital status, completion of obligatory service years, lack of established family, dissatisfaction with organizational decision-making, dissatisfaction with professional development opportunities, having a low continuous organizational commitment, and a non-conducive work environment were factors that increased turnover intention of healthcare professionals.

### Recommendations

Improving the working environment, continuous organizational commitment, and decision-making of the organizations, and increasing opportunities for professional development helps to reduce healthcare professionals' turnover intention.

## Data availability statement

The original contributions presented in the study are included in the article/Supplementary material, further inquiries can be directed to the corresponding authors.

## Ethics statement

The studies involving human participants were reviewed and approved by Salale University's Research and Ethical Review Committee. The patients/participants provided their written informed consent to participate in this study.

## Author contributions

The ideal conception, study design, data acquisition, analysis, and interpretation were all contributed to by all of the authors. All authors have reviewed the manuscript and approved its publication in this journal.
