# Supplementary material for: The magnitude of healthcare professionals' turnover intention and associated factors during the period of COVID-19 pandemic in North Shewa Zone government hospitals, Oromia region, Ethiopia, 2021
Source: Front Health Serv. 2022 Dec 8;2:918843. doi: 10.3389/frhs.2022.918843 (PMC10012650; doi:10.3389/frhs.2022.918843)
Supplement: Supplementary file 1 [file Data_Sheet_1.docx]

## Annex I: Questionnaires

**Part I. Socio-demographic**

| S/n | Variables | Responses |
| --- | --- | --- |
| 1 | Sex | 1. M 2. F |
| 2 | Age in years | ­­­­­­­ __________________ |
| 3 | Marital status | 1. Married 2. Single  3.Divorced 4.Widowed |
| 4 | Ethnicity | 1. Oromo 2. Amhara  3. Tigre 4. Others __________ |
| 5 | Religion | 1. Orthodox 2. Muslim  3. Protestant 4. Others_________________ |
| 6 | Educational qualification | 1. Diploma 2. BSc degree  3. Masters/specialty& above |
| 7 | Professional categories | 1. Nurse 2. Pharmacist  3. Midwifery 4. Medical doctor/ specialist  5. Laboratory 6. Others**___________________** |
| 8 | Have you upgraded/specialized? | 1.Yes 2.No |
| 9 | Years of work experience | _______________________ |
| 10 | Hospital type | 1. Primary 2. General |
| 11 | Did you complete your obligatory service year? | 1.Yes 2.No |
| 12 | Did you satisfy with your salary? | 1.Yes 2.No |
| 13 | Do you have an extra income source? | 1.Yes 2.No |
| 14 | Do you have an opportunity for other jobs | 1.Yes 2.No |
| 15 | Do you have established family | 1.Yes 2.No |
| 16 | Residence type | 1. Live with family  2. Live without family |

**Part II. Turnover intention related questions**

**Instruction:** Please put a thick mark (√) under the option you select.

| **S/n** | **Questions** | Strongly  disagree | disagree | No opinion | Agree | Strongly agree |
| --- | --- | --- | --- | --- | --- | --- |
| 1 | I intend to leave my organization |  |  |  |  |  |
| 2 | I intend to find another job over the next few months in other organizations |  |  |  |  |  |
| 3 | I often think about leaving this organization |  |  |  |  |  |

**Part-III: Organizational related factors**

|  | Satisfied with organization’s leadership style. | 1.Yes 2.No |
| --- | --- | --- |
|  | Satisfied with professional opportunity | 1.Yes 2.No |
|  | Satisfied with communication level of your organizations | 1.Yes 2.No |
|  | Satisfied with involvement in organizations’ decision making | 1.Yes 2.No |
|  | Satisfied with training opportunity in your organizations | 1.Yes 2.No |
|  | Satisfied with perceived organizational support | 1.Yes 2.No |

**Commitment related factors**

| **7** | **Affective commitment** | Strongly  disagree | Disagree | No opinion | Agree | Strongly agree |
| --- | --- | --- | --- | --- | --- | --- |
| 1 | I would be very happy to spend the rest of my career with this organization. |  |  |  |  |  |
| 2 | I really feel as if this organization's problems are my own. |  |  |  |  |  |
| 3 | I feel like a part of the family at my organization |  |  |  |  |  |
| 4 | I feel emotionally attached to this organization. |  |  |  |  |  |
| **8** | **Continuous commitment** |  |  |  |  |  |
| 1 | Right now, staying with my organization is a matter of necessity as much as a desire. |  |  |  |  |  |
| 2 | It would be very hard for me to leave my organization right now, even if I wanted to |  |  |  |  |  |
| 3 | Too much of my life would be disrupted if I decided to leave my organization. |  |  |  |  |  |
| 4 | One of the few negative consequences of leaving this organization would be the scarcity of available alternatives. |  |  |  |  |  |
| 9 | **Normative commitment** |  |  |  |  |  |
| 1 | Even it was to my advantage, I do not feel it would be right to leave my organization now. |  |  |  |  |  |
| 2 | I would not leave my organization right now because I have a sense of obligation to the people in it. |  |  |  |  |  |
| 3 | I owe a great deal to my organization |  |  |  |  |  |

**Part IV: Question on job related factors**

| **S/n** | **Job satisfaction related questions** | Strongly  Disagree | Disagree | **No**  **opinion** | **Agree** | **Strongly**  **agree** |
| --- | --- | --- | --- | --- | --- | --- |
| 1 | I feel fairly satisfied with my present job. |  |  |  |  |  |
| 2 | Most days I am eager about my work. |  |  |  |  |  |
| 3 | Each day of work seems like it will never end. |  |  |  |  |  |
| 4 | I find real enjoyment in my work |  |  |  |  |  |
| 5 | I consider my job rather pleasant. |  |  |  |  |  |
| **S/n** | **Job performance related questions** |  |  |  |  |  |
| 1 | My performance is better than that of my colleagues with similar qualifications. |  |  |  |  |  |
| 2 | I am satisfied with my performance because it is mostly good. |  |  |  |  |  |
| 3 | My performance is better than that of employees with similar qualifications in other organizations. |  |  |  |  |  |
| **S/n** | **Work environment related questions** |  |  |  |  |  |
| 1 | I feel uncomfortable to work in this organization. |  |  |  |  |  |
| 2 | I feel insecure in the working environment. |  |  |  |  |  |
| 3 | I experience unwelcome verbal and physical conduct from my boss. |  |  |  |  |  |
| 4 | I am not able to receive support from my boss, colleagues and juniors. |  |  |  |  |  |
| 5 | My relationship with colleagues and peers is not smooth. |  |  |  |  |  |
| 6 | Working environment of team is not good for career growth. |  |  |  |  |  |
| **S/n** | **Job stress related questions** |  |  |  |  |  |
| 1 | I often feel stress at work. |  |  |  |  |  |
| 2 | The job difficulty usually brings me sleeplessness |  |  |  |  |  |
| 3 | My job makes me nervous. |  |  |  |  |  |
| 4 | I feel exhausted after daily work |  |  |  |  |  |
| 5 | It’s helpful for my health if I change my job |  |  |  |  |  |
| 6 | I feel weak and dispirited at work |  |  |  |  |  |
| 7 | I feel more hot tempered at work |  |  |  |  |  |
| 8 | I feel depressed and unhappy at work |  |  |  |  |  |
| **S/n** | **Work overload related questions** |  |  |  |  |  |
| 1 | I experience excessive work pressure. |  |  |  |  |  |
| 2 | I work for long hours, on overtime and even on holidays. |  |  |  |  |  |
| 3 | I am unable to meet the demands of my job |  |  |  |  |  |
| 4 | I spend so long at work that my outside relationships are suffering. |  |  |  |  |  |
| 5 | I am so busy and I find difficult to concentrate on my job |  |  |  |  |  |
| 6 | I feel tired during the day due to excessive work load. |  |  |  |  |  |
